# Methods for comprehensive experimental identification of RNA-protein interactions

**DOI:** 10.1186/gb4152

**Published:** 2014-01-27

**Authors:** Colleen A McHugh, Pamela Russell, Mitchell Guttman

**Affiliations:** 1 Division of Biology and Biological Engineering, California Institute of Technology, Pasadena, CA 91125, USA

## Abstract

The importance of RNA-protein interactions in controlling mRNA regulation and non-coding RNA function is increasingly appreciated. A variety of methods exist to comprehensively define RNA-protein interactions. We describe these methods and the considerations required for designing and interpreting these experiments.

## Introduction

Over the past decade there has been an increasing appreciation of the importance of RNA-protein interactions in controlling many aspects of gene regulation [1,2]. The explosion in sequencing technologies has enabled exploration of the transcriptome at unprecedented depth [3]. This has led to a growing appreciation of the widespread role of alternative messenger RNA (mRNA) splicing [4-7], processing [8], editing [9-11] and methylation [12,13] in generating diverse mRNAs and in controlling the stability and translation of mRNA. Furthermore, this has led to the identification of diverse classes of non-coding RNAs (ncRNAs), including many thousands of long non-coding RNAs (lncRNAs) that resemble mRNA but are not translated into proteins [14-17].

The central role of RNA-protein interactions in controlling mRNA processing [1,2] and ncRNA function [18,19] is now clear. Many proteins are known to be required for various aspects of mRNA processing [1]. These include the ubiquitously expressed serine-rich (SR) proteins [20] and heteronuclear ribonucleoproteins (hnRNPs) [21], as well as the cell type-specific Nova [22], Fox [23] and Muscleblind [24] proteins, which all play important roles in the regulation of alternative splicing in different cell types [2,25,26]. Yet, precisely how these proteins control the diversity of cell type-specific mRNA remains largely unclear [2,27]. In addition, the proper cellular functions of virtually all ncRNAs - including those with catalytic roles [28,29] - depend upon the formation of RNA-protein complexes [18,19,30]. These include classical examples such as ribosomal RNAs, small nuclear RNAs and small nucleolar RNAs that control translation, splicing and ribosomal biogenesis, as well as small ncRNAs such as microRNAs and piwi-associated RNAs that control mRNA stability and translation [31], and silencing of DNA repeats [32]. In addition, lncRNAs play key functional roles in controlling cellular regulation [18,19,33-37], likely through their interactions with diverse classes of proteins [18,19]. To date, the full spectrum of proteins that interact with ncRNAs is still unknown [13,14].

The past decade has seen a strong interplay between method development, exploration and discovery about RNA biology. The methods for exploring RNA-protein interactions can be split into two general categories: ‘protein-centric’ and ‘RNA-centric’ methods. The protein-centric methods generally rely on the ability to purify a protein [38-40], or class of proteins [41], followed by sequencing of the associated RNAs to map RNA-binding proteins (RBPs) across the transcriptome at high resolution. Conversely, the RNA-centric approaches generally capture a given RNA [42-44], or class of RNAs [45,46], and identify the associated proteins using methods such as mass spectrometry (MS).

Protein-centric approaches have been widely used to generate binding maps of different RBPs across the transcriptome and have provided important insights into how mRNA processing is controlled in the cell [21,23,47,48]. These methods have also been used to gain initial insights into some of the proteins that can interact with lncRNAs [49-51]. Because these methods require knowledge of the protein, they are of more limited utility for defining the proteins that associate with a given RNA transcript. The RNA-centric methods have been more generally used to determine the complexes associated with a specific ncRNA in the cell. Indeed, the protein compositions of several classical ncRNA complexes, including those of telomerase RNA [42], small nuclear RNA [43], 7SK RNA [44] and RNase P [52], have been identified using these approaches.

In this review, we discuss approaches for identifying RNA-protein interactions and the challenges associated with interpreting these data. We describe the various protein-centric methods, including native and crosslinking-based methods, and explore the caveats and considerations required for designing, performing and interpreting the results of these experiments. We describe approaches that have been developed to account for analytical biases that can arise in these data. Furthermore, we describe the various RNA-centric methods for the identification of unknown RNA-binding proteins, including the various RNA tags used, purification schemes and detection methods. While conceptually simple, the RNA-centric methods are still not as common as the protein-centric methods because they require an extraordinary amount of starting material to purify enough protein required for detection [53]. We describe the challenges associated with these methods and their interpretation. Finally, we discuss the future steps that will be needed to synthesize the results of these complementary approaches and enable the systematic application of such methods to new classes of ncRNAs.

## Protein-centric methods to study RNA-protein interactions

The predominant methods for examining RNA-protein interactions are based on protein immunoprecipitation. These methods generally utilize antibodies to pull down the protein of interest and its associated RNA, which is reverse transcribed into cDNA, PCR amplified and sequenced [38,54-59]. Bioinformatic analysis is then used to map reads back to their transcripts of origin and identify protein binding sites [60,61].

**Figure 1:**
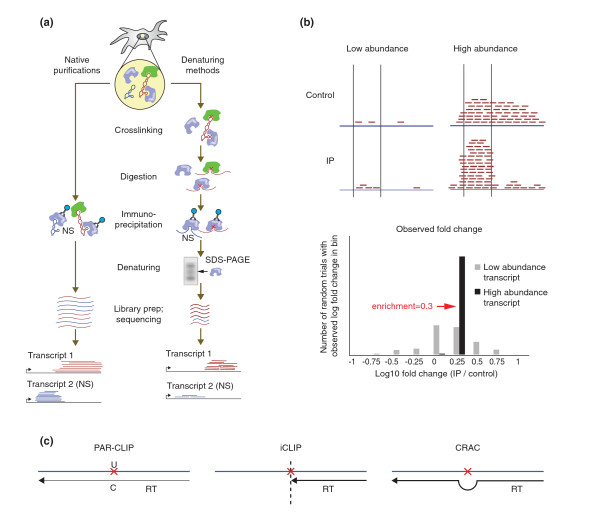
**Protein-centric methods for detecting RNA-protein interactions. (a)** Schematic of native and denaturing methods. RNA-protein crosslinks are represented by red Xs. Non-specific interactions in solution are labeled (NS) and represented by blue RNA fragments. **(b)** Computational considerations for identification of interaction sites. The top panel depicts two transcripts - one low-abundance and one high-abundance - that both contain a region that is twofold enriched in the immunoprecipitated (IP) sample over a control. Enrichment measurements in the low-abundance case suffer from high variance. The bottom panel shows simulated enrichment values in a low-abundance region and a high-abundance region, which both have a twofold enrichment in the IP sample. For the low abundance region, the observed log-fold changes are often far from the true underlying value while the abundant transcript shows a more consistent enrichment estimation. **(c)** A schematic of methods for mapping the precise protein binding sites on RNA. PAR-CLIP takes advantage of U → C transitions induced by UV crosslinking after 4SU incorporation. iCLIP uses the occasional arrest of reverse transcription at crosslink sites and tags and sequences these positions. CRAC relies on reverse transcription errors (deletions and substitutions) at crosslink sites to map sites. CRAC, cross-linking and analysis of cDNA; iCLIP, individual-nucleotide resolution cross-linking and immunoprecipitation; PAR-CLIP, photoactivatable-ribonucleoside-enhanced cross-linking and immunoprecipitation.

There are several variants of these methods, which can be broken down into two main classes: native [39,40,51,58,62-64] and fully denaturing purifications [22,55-57,59,65] (Figure 1a).

### Native purifications

Native purification methods, often known simply as RIP (RNA immunoprecipitation), purify RNA-protein complexes under physiological conditions. The advantage to these methods is that they preserve the native complexes present in the cell. Yet, these methods also have several limitations. The first and perhaps best described is due to the non-physiological formation of RNA-protein interactions in solution. Indeed, it has been shown that purification of an RNA-binding protein can retrieve RNAs, even when the RNA and protein are not present in the same cell type and therefore could not be interacting *in vivo*[66]. Furthermore, the RNAs that are purified are generally very well correlated with the abundance of the RNA, with ribosomal RNAs being the largest contaminating RNA species in virtually all protein purifications [38]. As a consequence, specific interactions that occur with low abundance transcripts may be masked by non-specific interactions that occur with highly abundant transcripts [38].

Because of these issues, there has been some controversy about the nature of the interactions detected by these methods. For example, many lncRNA-protein interactions have been explored using native purifications of proteins such as those found in Polycomb repressive complex 2 (PRC2) [51,58,62]. In these studies, a very large percentage of lncRNAs, as well as mRNAs, were identified as interacting with PRC2 [58], with a recent study arguing that virtually all transcripts interact with PRC2 in the cell [62]. This has led to debate over the biological significance of lncRNA-PRC2 interactions, with some arguing that they are simply non-specific interactions [67]. However, it is clear that at least some lncRNAs interact with PRC2 [49,50,68] and that these interactions have clear functional roles [58,69,70]. While it is clear that both native and denaturing purification methods can identify a similar core set of functional interactions [71], the extent to which non-specific interactions are also identified by the native methods remains unclear. As such, interactions identified using native purification methods often require further experimental validation, such as through the integration of multiple distinct experimental approaches [49,71,72].

### Denaturing methods for RNA-protein interactions

To account for these concerns, denaturing methods were introduced. By crosslinking RNA-protein complexes in the cell and purifying the complex under denaturing conditions, one can distinguish *in vivo* interactions that are crosslinked in the cell from interactions that form subsequently in solution.

The dominant method for crosslinking RNA-protein complexes is treatment of cells with short wavelength UV light to create a covalent linkage between physically interacting RNA and protein molecules in the cell, but not between interacting proteins [73]. Methods such as crosslinking and immunoprecipitation (CLIP) purify an RNA-protein complex using stringent wash conditions followed by denaturation of all complexes by heating in sodium dodecyl sulphate (SDS), running the samples on an SDS-polyacrylamide gel electrophoresis (PAGE) gel, and extracting the crosslinked RNA-protein complex, which will run at a size slightly larger than the protein itself, from the gel [74,75]. The main limitation of this method is the low efficiency of UV crosslinking. To account for this, a variant that significantly increases crosslinking efficiency while retaining the main features of UV crosslinking was introduced: photoactivatable-ribonucleoside-enhanced (PAR)-CLIP [56]. This approach incorporates a nucleotide analog (such as 4′-thiouracil) into cells, followed by treatment of the cells with long-wavelength UV. The drawback to this approach is that it is only amenable to cells in culture and cannot be applied to primary tissues.

A significant concern with using UV crosslinking methods is that they may miss real RNA-protein interactions simply because they are not efficiently captured by UV crosslinking. Indeed, several RBP families that do not directly interact with nucleic acid bases but instead interact with other features, such as the sugar phosphate backbone, have been shown to have lower crosslinking efficiency with UV [76]. Because UV-induced crosslinking is still incompletely understood at the biophysical level [38], it is unclear which types of interactions might be missed or what frequency of real interactions may be missed. In addition, because UV only crosslinks direct RNA-protein interactions, it will not capture interactions that occur through a complex of multiple proteins. As an example, interactions with many chromatin regulatory proteins have proven challenging to identify by purification under denaturing conditions after UV crosslinking, likely because the precise protein that interacts directly with RNA is still unknown [33].

Other crosslinking methods, such as formaldehyde, can eliminate the need to know the directly interacting protein, but alternative denaturing strategies are needed since purification from a denaturing SDS-PAGE gel would not resolve at the size of the protein. An alternative approach that leverages many of the conceptual features of the CLIP method is to utilize direct denaturing conditions rather than separation through an SDS-PAGE gel. These methods use affinity tags coupled to the protein of interest for capture by purification in denaturating conditions (that is, using urea or guanidine) [59-65]. The advantage to this approach is that it can be used with any crosslinking protocol, including formaldehyde crosslinking, which otherwise could not be separated on an SDS-PAGE gel [59]. Yet, this approach requires the ability to express a tagged version of the RBP of interest in the cell.

### Analysis of protein-centric RNA-protein interaction data

There are two primary goals in the analysis of protein-centric experiments: defining which RNAs are bound by the specific protein and defining the specific protein-binding sites on these RNAs.

It is important to compare the sample to a negative control since observing reads from a specific RNA alone may not be indicative of a real interaction. One control is to normalize the coverage level of an RNA observed after purification to its abundance in total lysate. Yet, this control only accounts for issues due to RNA abundance: interactions can occur due to association with the purification resin or other features of the system. To account for this, other proteins can be used as negative controls. However, the negative control should be selected with care, as a non-RNA-binding protein is likely to have lower non-specific RNA binding. Indeed, simply mutating the RNA-binding domain of a protein has been shown to remove both specific and non-specific interactions formed by a protein [77]. The ideal control is to demonstrate that the interaction is not present in the absence of crosslinking [22,38]. However, this control can only be used in conjunction with a fully denaturing protocol.

Furthermore, comparing the sample with a negative control requires proper statistical methods because the inherently low denominator for low abundance RNAs will lead to a higher variance in the enrichment measurement (Figure 1b). This challenge is similar to the problems faced when computing differential expression using RNA-Seq data [78], and many different statistical solutions, including parametric, non-parametric and permutation methods, have been proposed [79-81].

The second goal is to map protein-binding sites on RNA. A major consideration is the size of the RNA after digestion. While in theory the ideal size is that of the protein footprint itself, several considerations favor slightly larger sizes. One issue is the alignability of the sequencing reads, as very small fragments may not be able to be uniquely aligned to the transcriptome. Another concern is that overdigestion may lead to a loss of real binding sites by preferentially eliminating certain protein footprints [75].

Because UV-crosslinking is irreversible, reverse transcription can halt at the site of crosslinking even after protein removal [22,25]. While this was originally considered a disadvantage of UV crosslinking, it has been successfully used by several methods, including the CLIP variant individual-nucleotide resolution CLIP (iCLIP), to identify protein-binding sites on RNA with improved resolution [55,57]. In addition to RT stops, crosslink sites also show a higher rate of deletions and mismatches - these have also been used to identify binding sites [61] (Figure 1c). Yet, great care must be exercised when interpreting these RT-induced stop sites and errors, as RNA damage due to UV light is also known to inhibit reverse transcription [82].

## RNA-centric methods

Protein-centric methods are of limited utility for identifying novel RBPs that interact with a specific RNA or for the characterization of novel classes of ncRNAs for which the identities of the RNA-binding proteins are still unknown. An alternative approach is to use an RNA-centric protein identification strategy. The general idea is simple: rather than using an antibody to capture a protein of interest and sequencing the associated RNA, these methods purify an RNA of interest and identify the associated protein complexes, using methods such as MS. We will explore the different variants of these methods below, focusing on those designed to comprehensively identify novel RNA-protein interactions.

### RNA affinity capture methods

One general approach to capture RNA is to exploit naturally occurring interactions between RNA and protein - such as the bacteriophage MS2 viral coat protein, which binds tightly to an RNA stem loop structure [83]. In this approach, repeats of the MS2-binding RNA stem loop are appended to an RNA of interest and the tagged RNA complex is purified by coupling the MS2 protein to a solid support or resin [84-86]. These dual component interactions can be optimized to enable increased affinity and stability [44,87]. As an example, a recent approach makes use of an engineered Csy4 protein, a component of the bacterial clustered regularly interspaced short palindromic repeats (CRISPR) system, to generate a tag with higher affinity than can be achieved for traditional RNA tags, including MS2 and PP7 [87]. Alternatively, artificially designed RNA aptamers can be developed and selected for binding to commonly used resin-conjugated proteins [43,88]. An example of this is the S1 aptamer that binds to streptavidin [89,90].

The differences between these methods can be exploited when trying to elute their respective RNA-protein complexes. In general, protein complexes are eluted from a support resin by boiling in SDS [87]. This approach will dissociate bound material from the resin, including complexes bound specifically through the tag and those bound non-specifically directly to the resin. For several of these affinity tags, complexes can be eluted more specifically. For example, in the case of the S1 aptamer, the weaker affinity of the S1-streptavidin interaction compared with the biotin-streptavidin interaction can be exploited to enable specific elution of the RNA through a competitive approach using high concentrations of biotin [91]. In the CRISPR system, because of the nature of the Csy4 mutant used, one can specifically cleave the complex through the addition of imidazole. Indeed, the specificity of elution dramatically increases the specificity of the purified complexes and can improve detection sensitivity [87].

### Purification of RNA and associated protein complexes

**Figure 2:**
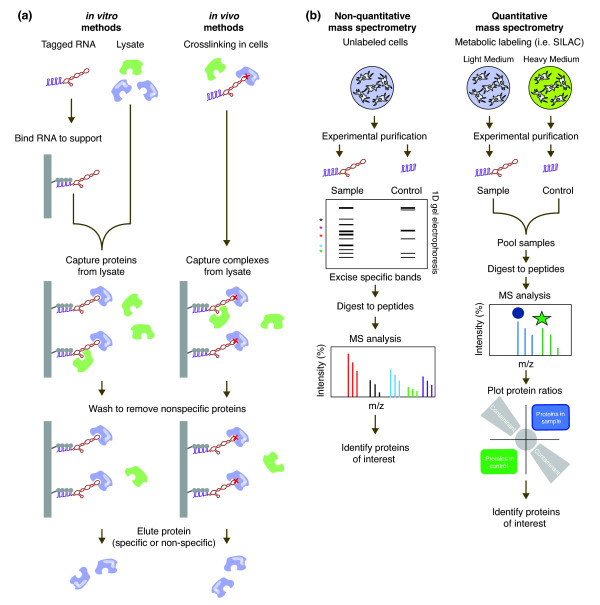
**RNA-centric methods for the purification and identification of RNA-binding proteins. (a)** Examples of purification schemes for RNA-binding proteins using *in vitro* and *in vivo* approaches. For *in vitro* approaches, a tagged RNA construct is generated and bound to a solid support. In this example the MS2 protein-RNA interaction tagging method is shown with the target RNA (red), MS2-binding motif (purple) and MS2 protein (gray). Cell lysate is prepared and proteins from lysate are captured using the tagged RNA *in vitro*. For *in vivo* approaches, the target RNA is crosslinked to specific interacting RNA-binding proteins in living cells using UV, formaldehyde or other cross-linkers. Cells are lysed and the RNA-protein complexes captured from solution. In both scenarios, the complex is washed to remove non-specific interactions (green proteins). Finally the bound proteins are eluted. **(b)** MS is commonly used to identify the RBPs in a purified sample. In non-quantitative MS approaches, RBPs are purified from unlabeled cell material using either an RNA of interest or a control construct. After separation by one-dimensional gel electrophoresis, specific protein bands from the sample are selected, excised and identified by MS analysis. In quantitative MS approaches, proteins are differentially labeled based on their initial cell populations. Experimental and control purifications are performed on these labeled populations and the purified RBPs are pooled to create a single sample. MS analysis allows direct comparison of labeled peptides, which can then be quantified to determine specific proteins in the sample compared with the control. SILAC, stable isotope labeling by amino acids in cell culture.

RNA-centric approaches can be grouped into one of two major classes: *in vitro* and *in vivo* purification methods (Figure 2a). The *in vitro* approaches generally employ a synthetic RNA bait to capture and identify proteins from cellular extracts [43,88,90]. In contrast, the *in vivo* approaches capture the RNA-protein complexes present in the cell [45,46,85,92]. While the *in vivo* methods preserve the context of true RNA-protein interactions, they are more technically challenging, especially if the target RNA is of low abundance in the cell.

Similar to the protein-centric methods, purification of RNA under native conditions can lead to re-association or formation of non-specific RNA-protein interactions in solution. Studies using *in vitro* approaches or performing purifications in native conditions have generally found association between the RNA of interest and highly abundant proteins in the cell, such as hnRNPs [85,91,92]. Whether these represent real biological interactions or non-specific associations is unclear because only a handful of RNAs have been purified to date. To address this, a recent study made use of UV crosslinking and purified RNA complexes under fully denaturing conditions (using 8 M urea), which will only capture *in vivo* crosslinked complexes [85]. Using this approach, there were clear differences in the proteins identified after purifications performed in native and denaturing conditions. DNA-binding proteins and other abundant nucleic acid-binding proteins were present only in the native purification, but not in the denaturing purification, suggesting that at least some of these purified proteins might be due to non-specific association in solution. Other approaches use stringent, high-salt wash conditions to reduce non-specific interactions during RNA-protein complex purification [45,93,94].

The challenge with denaturing approaches is that they require complexes to be crosslinked in the cell, which is not efficient. In addition, several crosslinking strategies, such as formaldehyde crosslinking, may have additional technical challenges associated with the identification of crosslinked peptides by MS [95].

### Defining the proteins that associate with an RNA

We will focus on MS methods for identification of RNA-binding proteins. There are two principal ways that have been used to comprehensively identify these protein complexes by MS: non-quantitative and quantitative MS (Figure 2b).

In the non-quantitative methods, purified proteins from the RNA sample of interest and a control are separated by gel electrophoresis and stained for total protein. Protein bands that are present only in the sample of interest but not the control are extracted and the proteins identified by MS [84]. Alternatively, the total proteome can be analyzed by MS to detect all proteins purified in a sample [87,96]. The advantage to the latter approach is that all proteins can be identified in the sample, including those that are not visible on the gel. In this approach, the control can also be analyzed to identify non-specific proteins for exclusion. However, it is difficult to directly compare the quantities of proteins identified in the sample and control, due to variations in the relative intensity of identified peptides in independent runs [53].

To overcome this limitation, one can use quantitative MS to simultaneously compare the proteins in the sample and control. There are several ways to do this (reviewed in [53]). In one popular method used for RNA-protein analysis, cells are metabolically labeled to generate differentially tagged protein pools for MS analysis, in which the isotopes of the proteins are compared to provide direct quantification [97]. The advantage to this approach is that the ratios of peptides from the experimental and control samples can be directly compared to allow discrimination of true binding partners from non-specific interactors. This method can account for some of the issues associated with abundant protein association. As an example, in quantitative MS experiments, most of the abundant proteins, such as hnRNPs, show equal abundance in both experimental and control samples, suggesting that these interactions are not specific to the RNA of interest [91].

The choice of which MS approach to use for the identification of RBPs depends on the nature of the upstream purification. When utilizing a protocol where the resulting protein purification yields little background in the control sample, a non-quantitative approach may work well. The CRISPR-Csy4 system, for instance, was previously shown to enable very high stringency and specific elution, and because of this a non-quantitative approach provided reliable results [87]. Similarly, when employing crosslinking followed by a denaturing purification strategy, non-quantitative MS might provide a good approach. In contrast, when using a system with higher background, a quantitative MS approach can provide increased ability to discriminate between specific and non-specific binders.

### Analytical challenges with RBP MS analysis

There are several analytical challenges for identifying proteins associated with an RNA by MS. Similar to the protein-centric methods, great care must be taken to select informative negative controls for the RNA-centric methods. Controls that are often used include a different cellular RNA [92], sequences lacking known protein-binding structures [85-91], tag-only controls [44], antisense RNA [71,98] or non-specific RNA sequences [99]. In these cases, any non-specific protein interactions due to abundance, nucleic acid binding or the tag itself should be identical for the target RNA and controls. However, the ideal negative control is not clearly established because there may be some specific features of the RNA of interest that bind non-specifically to certain proteins. In cases where protein-RNA crosslinking is employed, the ideal control would be a non-crosslinked sample because it represents the identical purification of the same RNA but without any *in vivo* crosslinked complexes [96]. However, this approach requires the use of *in vivo* crosslinking followed by a denaturing purification and therefore is not applicable to all purification methods. In the absence of this, several different negative controls should be included to ensure robustness of the results identified.

A significant challenge in the identification of unknown RBPs is the generation of sufficient material for MS, particularly for low abundance RNA-protein complexes. Unlike sequencing methods that enable nucleic acid amplification, the amount of protein purified in these experiments cannot be amplified. For this reason, RNA-centric methods have mostly been applied to highly abundant RNAs, such as 7SK [44], snRNPs [100], Let-7 [99] and IRES [85]. More recently, these approaches have been used to define proteins associated with all mRNA by UV crosslinking RNA-protein complexes, capturing polyadenylated transcripts using oligo-dT coupled magnetic beads, and detecting associated proteins by quantitative MS [45,46,94]. Yet, application of this approach to identify binding partners of individual mRNAs, lncRNAs or other low abundance RNAs is still a significant challenge.

## Future directions

While much work has been done to develop methods to identify and examine RNA-protein interactions, there are still significant challenges that need to be addressed. To date, we still do not know the protein complexes that interact with most RNAs in the cell - including mRNAs, classical ncRNAs and lncRNAs. For lncRNAs in particular, we know little about the diversity of proteins that they may interact with. Many of the protein complexes that have been identified to interact with lncRNAs do not fall into traditional RNA-binding protein classes, making it difficult to generate accurate predictions of what these complexes may look like. Understanding the protein complexes that interact with lncRNAs will be an important first step toward understanding their various biological functions and mechanisms. The major challenge with defining these proteins is that the RNA-centric methods are still not well suited for exploring low abundance transcripts. Future work will be needed to address this challenge and to define the protein complexes that interact with a given lncRNA or individual mRNA.

Although the development of the protein-centric and RNA-centric approaches has mostly proceeded independently, we can now begin to combine the results of these complementary approaches to build a complete picture of the repertoire of RBPs in a cell and define their roles in binding and modulating the functions of various classes of RNA [101]. Several recent studies have begun to examine protein binding at a transcriptome-wide scale [45,94,102-106]. In these studies, RBPs [45,94,102,104] and/or their binding sites [45,94,102-106] have been identified by MS or high-throughput sequencing, respectively. By exploring the different components of RNA-protein complexes, we will be able to identify new RBPs, as well as discriminate the timing of the binding of a set of given RBPs to an individual RNA [107]. This will ultimately provide a more complete understanding of the function of RNA-protein complexes, including how these complexes assemble and how they modulate cellular function.

## Abbreviations

CLIP: Crosslinking and immunoprecipitation; CRISPR: Clustered regularly interspaced short palindromic repeats; hnRNP: Heterogenous ribonucleoprotein; lncRNA: Large non-coding RNA; MS: Mass spectrometry; ncRNA: Non-coding RNA; PAR: Photoactivatable-ribonucleoside-enhanced; PCR2: Polycomb repressive complex 2; RBP: RNA-binding protein.
